# HSP-72 Accelerated Expression in Mononuclear Cells Induced *In Vivo* by Acetyl Salicylic Acid Can Be Reproduced *In Vitro* when Combined with H_2_O_2_

**DOI:** 10.1371/journal.pone.0065449

**Published:** 2013-06-06

**Authors:** Alvaro A. Sandoval-Montiel, Martha Zentella-de-Piña, José L. Ventura-Gallegos, Susana Frías-González, Ambar López-Macay, Alejandro Zentella-Dehesa

**Affiliations:** 1 Departmento de Medicina Genómica y Toxicología Ambiental, Instituto de Investigaciones Biomédicas, Universidad Nacional Autónoma de México, México, D.F., México; 2 Unidad de Bioquímica, Instituto Nacional de Ciencias Médicas y Nutrición Salvador Zubirán, México, D.F., México; 3 Departmento de Bioquímica, Facultad de Medicina, Universidad Nacional Autónoma de México, México, D.F., México; 4 Laboratorio de Líquido Sinovial, Instituto Nacional de Rehabilitación, México D.F., México; University of South Florida College of Medicine, United States of America

## Abstract

**Background:**

Among NSAIDs acetyl salicylic acid remains as a valuable tool because of the variety of benefic prophylactic and therapeutic effects. Nevertheless, the molecular bases for these responses have not been complete understood. We explored the effect of acetyl salicylic acid on the heat shock response.

**Results:**

Peripheral blood mononuclear cells from rats challenged with acetyl salicylic acid presented a faster kinetics of expression of HSP-72 messenger RNA and protein in response to *in vitro* heat shock. This effect reaches its maximum 2 h after treatment and disappeared after 5 h. On isolated peripheral blood mononuclear cells from untreated rats, incubation with acetyl salicylic acid was ineffective to produce priming, but this effect was mimicked when the cells were incubated with the combination of H_2_O_2_+ ASA.

**Conclusions:**

Administration of acetyl salicylic acid to rats alters HSP-72 expression mechanism in a way that it becomes more efficient in response *to in vitro* heat shock. The fact that *in vitro* acetyl salicylic acid alone did not induce this priming effect implies that *in vivo* other signals are required. Priming could be reproduces in vitro with the combination of acetyl salicylic acid+H_2_O_2_.

## Introduction

The response of all eukaryotic organisms to thermal, environmental, and physiological stress involves the rapid production of a group of proteins denominated Heat shock proteins (HSPs) [1]. At the molecular level, the heat-shock response is a transient reprogramming of cellular activities featuring robust synthesis of HSPs, concomitant with cessation of normal protein synthesis [2]. HSPs are highly conserved Adenosine triphosphate (ATP)ases, which are ubiquitous and abundant in nearly all sub-cellular compartments. They are divided into different families according to their molecular size (i.e., HSP-100, -90, -70, -60, -40, and small HSPs). Among them, HSP-72 is the first to be express and consequently serves as an indicator of the speed and the strength of the heat shock response (HSR).

In mammalian cells, HSPs function as molecular chaperones and are essential for correct folding, assembly, and intracellular translocation of proteins, as well as for cell signaling. Some HSPs have constitutive forms, such as HSP-90 or -73, while other forms are inducible, such HSP-72 [3]. At the transcription level, expressions of the inducible forms of HSPs in the adult are orchestrated by the Heat shock transcription factor (HSF-1) [4].

The heat shock response has been described as an ordered genetic response to diverse environmental and physiological stressors that results in the immediate induction of genes encoding molecular chaperones, proteases, and other proteins that confer protection and facilitate recovery from cellular damage associated with the expression of misfolded proteins [5].

Recently, several pharmacological compounds that can induce or enhance induction of HSP synthesis have been identified, among them Non-steroidal anti-inflammatory drugs (NSAIDs) have been found to potentiate heat shock response [6], [7]. These inductive effects of NSAIDs have been associated to the activation of HSF1 [8], [9], [10], [11], [12]. Administration of ASA *in vivo* protects against oxidative damage and elicits other physiological benefits; however, the biochemical and molecular mechanisms involved in these effects are not completely understood [13], [14], [15]. Previous reports from our group have found that *in vivo* ASA treatment reverts some biochemical markers of oxidative damage [13], suggesting the activation of a general cytoprotective mechanism. Indomethacin treatment followed by heat shock in HeLa cells activates HSF1 and confers cytoprotection against lethal heat shock [7].

There is evidence that other NSAIDs, such as naproxen, can cause oxidative stress [16]. In addition, it has been shown that ASA (l0 µM) can generate H_2_O_2_ in rat adipose tissue, reaching a final concentration of 1 µM (unpublished personal communication from Zentella de Piña M, (May 2009)). It has also been reported that orogastric administration of ASA provides cytoprotection of rat lymphocytes against oxidizing agents [13], reversing some indicators of oxidative stress such as thiobarbituric acid reactive substances and protein carbonylation. Here, we have shown that the orogastric administration of ASA (45 mg/kg of body weight) to rats can prime isolated peripheral blood mononuclear cells (PBMCs) for faster kinetics of Heat Shock induced HSP-72. We also describe that *in vitro* incubation of PBMCs with H_2_O_2_ (5 µM) in combination with ASA (10 µM) can reproduce the accelerated expression of HSP-72 observed in PBMCs of ASA-treated rats.

## Results

**Figure 1:**
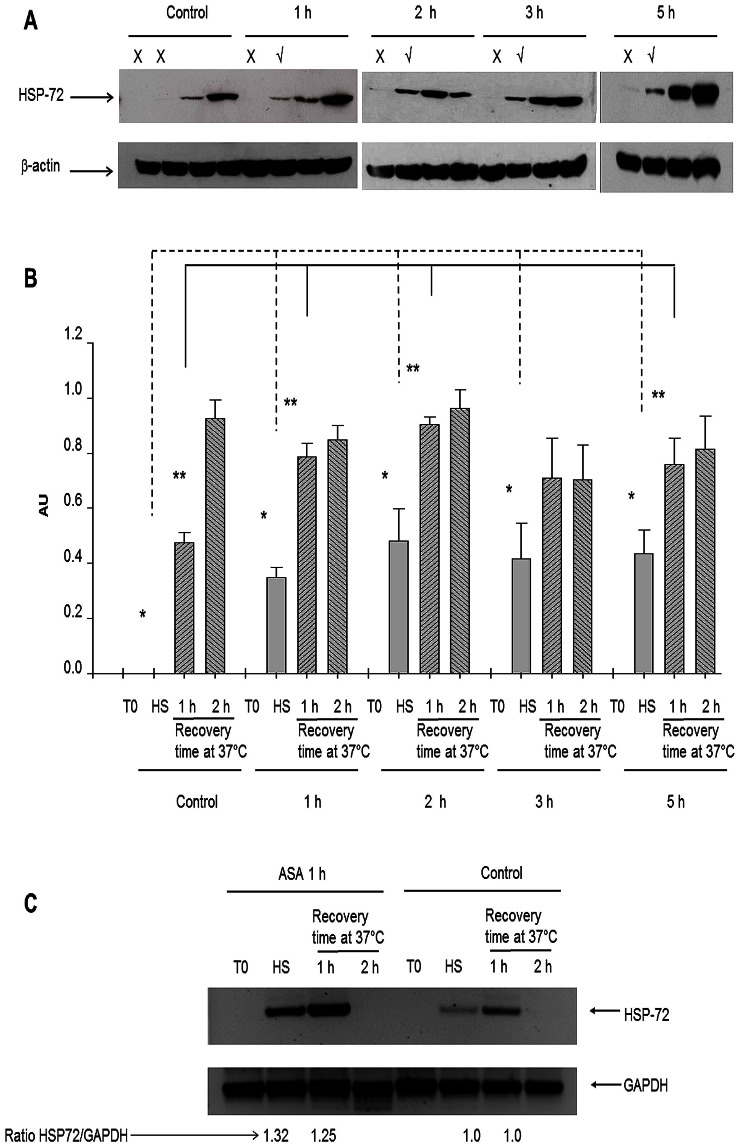
Time course of changes in HSP-72 expression before (T0), immediately after heat shock (HS) and 1 (1 h) and 2 (2 h) hours after recovery at 37°C in peripheral blood mononuclear cells (PBMCs) from rats, treated with or without acetylsalicylic acid (ASA 45 mg/kg of body weight). Cells were isolated 1, 2; 3, or 5 h after administration of ASA and subjected to heat shock protocol. Cells from untreated animals served as controls. 100 µg of total protein was placed per lane. A) Representative Western blots corresponding to cells from control animals or from rats challenged with ASA for 1, 2, 3, or 5 h. B) Histogram of the average signals expressed as Arbitrary Units (AU = OD for HSP-72/OD for β-actin) from three independent experiments. *Comparison of HSP-72 content immediately after heat shock between cells from ASA-challenged rats (for 1, 2, 3, or 5 h) or without it (control) (*n* = 3) (*p*<0.05). **A similar comparison 1 h after heat shock (*n* = 3) (*p*<0.05). *t*-student test was used. C) RT-PCR for HSP-72 mRNA content in PBMCs from rats with or without ASA treatment. PBMCs were obtained 1 h after ASA challenged and subjected to in vitro to heat shock. Total RNA was obtained immediately after heat shock. Products of RT-PCR were resolve in agarose gels. The numbers indicate the normalized band intensity (HSP-72/GAPDH).

When the content of HSP-72 in PBMCs from rats challenged with ASA (45 mg/kg bw) was evaluated no detectable expression of HSP-72 was observed, lanes marked with the X in Figures 1A and 1B. In all cases, the PBMCs were subjected to a heat shock (42°C for 45 min). HSP-72 content was assessed immediately after heat shock (HS) or 1 or 2 h later at 37°C. Immediately after HS, HPS-72 was detected only in cells from ASA challenged rats (lanes marked with √). When we compared HSP-72 expression 1 h after heat shock 70% higher content was observed compared to control cells 1 h after heat shock. This increased remained after 2, 3, and 5 h ASA challenge (83, 52, and 61% respectively). These results indicate that ASA administration *in vivo* primes HSP-72 expression in PBMCs subjected to an *in vitr*o heat shock.

HSP-72 mRNA content was studied through RT-PCR in cells from challenged animals following *in vitro* HS. Amplification products, were detected only from heat shock-subjected cells (Figure 1C), both in cells from control animals and those treated with ASA. However, in ASA-treated cells, the amount of HSP-72 PCR product immediately after HS was 65% higher compared with that found in control cells. These results indicated that the priming effect can also be observed at mRNA level.

**Figure 2:**
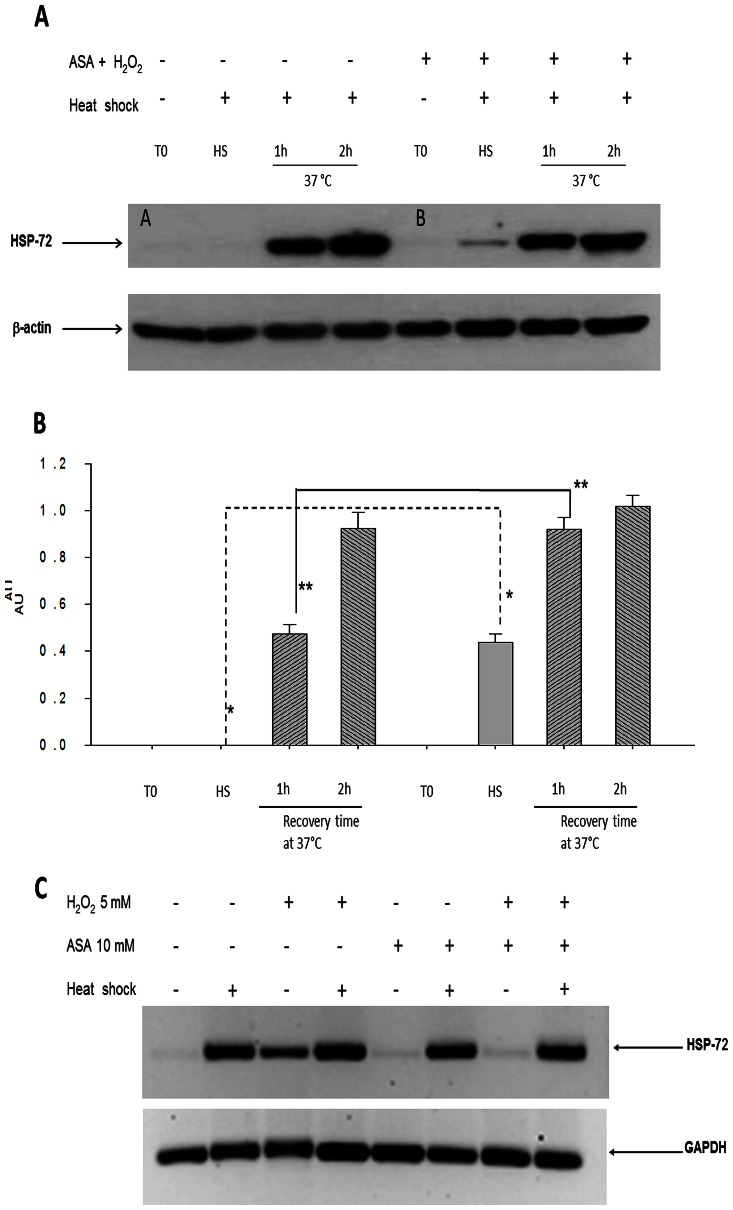
Time course of changes in HSP-72 expression before (T0), immediately after heat shock (HS) and 1 (1 h) and 2 (2 h) hours after recovery at 37°C in peripheral blood mononuclear cells (PBMCs) from untreated rats incubated with or without the combination ASA (10 µM)+H_2_O_2_ (5 µM). A) Western blots of HSP-72 from rat PBMCs (100 µg of total protein) incubated with (+) or without (-) the indicated treatments for 1 h before heat shock, β-actin was used as loading control. Protein extracts were obtained before *in vitro* incubation (T0), or immediately after Heat shock (HS) or (1****h) and 2 (2****h) hours after recovery at 37°C. B) Histogram of the average signals expressed as Arbitrary units (AU) = OD for HSP-72/OD for β-actin from three independent experiments. *Comparison of HSP-72 content immediately after heat shock between cells without any treatment an those incubated with the combination ASA+H_2_O_2_ (*n* = 3) (*p*<0.001). **Comparison of cells incubated 1 h after heat shock without any treatment an those incubated with the combination ASA+H_2_O_2_ (*n* = 3) (*p*<0.001). C) RT-PCR of HSP-72 mRNA from PBMCs treated *in vitro* with the combination ASA+H_2_O_2_ following heat shock. Total RNA was obtained immediately after heat shock. Products of RT-PCR were resolve in agarose gels.

Next we compared the priming effect of the ASA+H_2_O_2_ combination applied *in vitro* to that observed with the priming effect induced *in vivo* on PBMCs from ASA-challenged animals (45 mg/kg). The most significant effect was the accelerated expression of HSP-72 following *in vitro* heat shock when cells were pretreated with the ASA+H_2_O_2_ combination (lane 6 compared with lane 2 in Figure 2A). This accelerated expression of HSP-72 continued to be observed after 1 h following heat shock and disappearance after 2 h (Figure 2B).

We followed HSP-72 mRNA content in PBMCs incubated with the ASA+H_2_O_2_ combination following *in vitro* heat shock using RT-PCR. HSP-72 amplification products were detected only from cells subjected to heat shock, when cells were pre-incubated with the combination (Figure 2C). These experiments indicates that the priming effect on HSP-72 expression following heat shock can be induced in* vitro* when PBMCs are pre-incubated with ASA+H_2_O_2_ and requires heat shock for its transcriptional activation.

**Figure 3:**
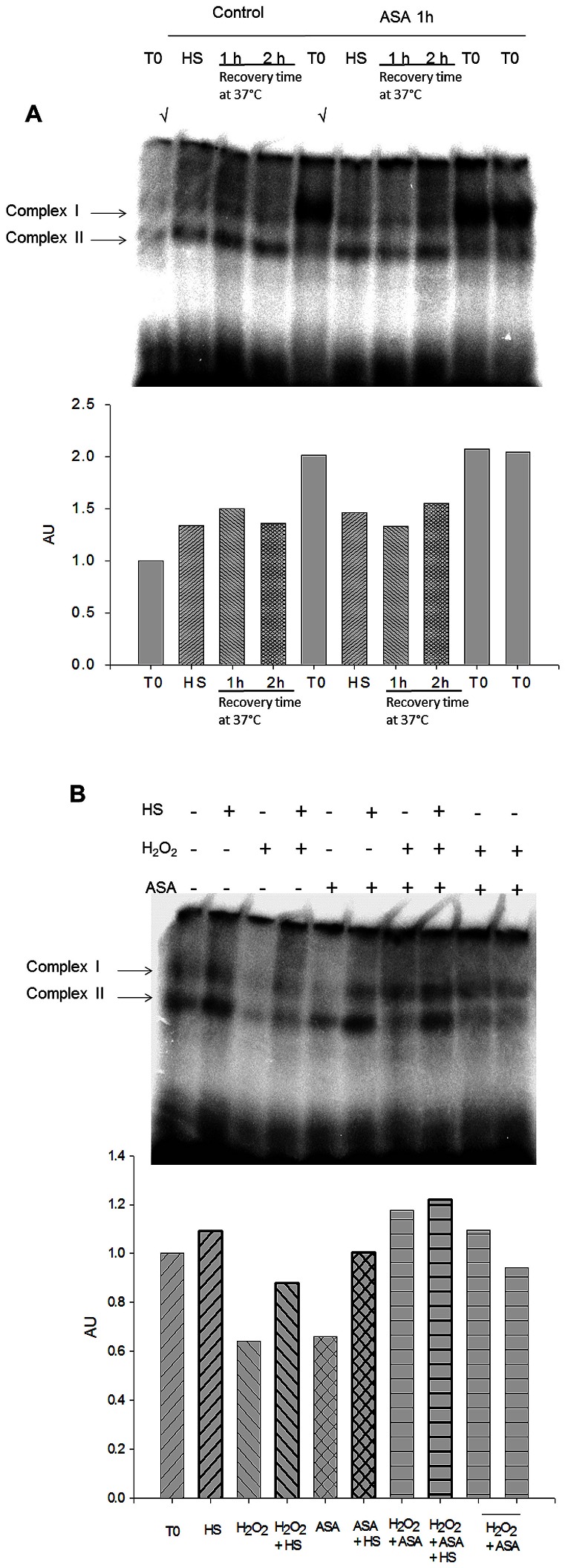
Time course of EMSAs of HSF1 before (T0), immediately after heat shock (HS) and 1 (1 h) and 2 (2 h) hours after recovery at 37°C from PBMCs. Nuclear extract protein was obtained and EMSA was performed with 10 µg of protein as described in the Materials and Methods. A) Nuclear extracts from PBMCs from control (Control) or ASA challenged rats (ASA 1****h) were analyzed at the indicated time points. B) Nuclear extracts from PBMCs from control animals received the indicated treatments *in vitro* 1 h before heat shock. DNA/protein complexes are labeled as: I and II. Histograms represent the relative amount of complex I under the different experimental conditions, normalized against control obtained at T0, both *in vivo* and *in vitro*.

We evaluate the presence of the transcription factor HSF-1 in the nucleus of ASA-challenged rat PBMCs by EMSA analysis. Figure 3A shows the result of *in vivo* experiments. Cells from control animals that received no ASA treatment (Control) show no DNA binding activity before heat shock, however, following heat shock a DNA/protein complex (Complex I) of moderate intensity was observed which was sustained for the next 2 h at 37°C. A strong signal for this DNA/protein complex was present in nuclear extracts from PBMCs from rats challenged with ASA for 1 h (lane mark with √), compared with control. It is important to note that this increase in HSF-1/DNA-binding activity was induced without the need of heat shock. At the end of heat shock DNA the binding activity fell and remained unchanged for the next 2 hours later at 37°C. We conclude that ASA treatment *in vivo* promotes DNA binding capacity of HSF-1 to DNA.

Since the combination ASA+H_2_O_2_ led to a faster expression of HSP-72 *in vitro* in response to heat shock, we evaluated the effect of this combination on HSF-1/DNA binding activity (Figure 3B). AS expected following heat shock (HS) we observed a small increase in the signal of complexes I and II (lanes 1 and 2). H_2_O_2_ led to decrease in the signal of both complexes under unstimulated conditions and also decreased the signal following heat shock (lanes 3 and 4). ASA alone led to a decrease of the signal of complex I but did not affect complex II (lane 5) and had no significant effect on the increased signal of complex I following heat shock (lane 6). While the combination of ASA+H_2_O_2_ produced a small increase in the signal of complex I (lane 7), the strongest signal of this complex was observed with the combination of heat shock+ASA+H_2_O_2_ corresponding to a 20% increase with respect to the untreated control (lane 8). Consequently, this result leads us to conclude that the combination of ASA+H_2_O_2_ *in vitro* promotes a very weak DNA-binding activity of HSF-1. It should be noted that the changes in complex II not always reflect the behavior of complex I.

## Discussion

The wide clinical benefit of ASA’s interference with inflammation and provision of analgesic effects has been extended to prevention of cardiovascular diseases, sustained pregnancy, and reduction in the risk of development of Alzheimer disease and colon cancer [14], [15], [16], [17], [18]. In addition to interference with cyclo-oxygenases I and II, ASA has been shown to modulate Nitric oxide (NO)-synthase, NF-kB signaling, and ATP metabolism, and to induce generation of H_2_O_2_, oxidative stress, and apoptosis. On the other hand, overexpression of HSP-72 exerts a cytoprotective effect in a variety of cellular systems [19], [20], [21]. In this study, we describe a link between ASA treatment and the efficiency of HSP-72 expression following heat shock in PBMCs in an *in vivo* model. This priming effect could be mimicked *in vitro* with the combination of ASA (10 µM) and H_2_O_2_ (5 µM) followed by heat shock.

Previous studies performed *in vitro* have shown that NSAIDs such SS and Indomethacin can activate DNA binding activity of HSF-1 in tumor cell lines such as HeLa cells without an increased in HSF1-dependent transcription of HSP-72 at supra-pharmacological concentrations (SS from 2 to 30 mM and Indomethacin from 100 to 1000 µM) [6], [7], [11], [12] our results confirm these effects of ASA on HSF-1 activation without transcription activity. We extend the implications of this response by describing it in primary cell (PBMCs) both *in vivo* and *in vitro*, in addition our model responded at pharmacological doses of ASA (10 µM). Furthermore we were able to mimic this effect *in vi*tro on PBMCs by combining heat shock ASA with H_2_O_2_. Considering that i*n vivo*, PBMCs are a primary target of pharmacological doses of ASA administration, our experimental model could serve to further explorer the *in vivo* mechanism linked to cytoprotection and other benefic effects of ASA and NSAIDs in general.

### Experiments *in vivo*

Priming of HSP-72 expression installed *in vivo* on PBMCs by ASA administration reached a maximal effect 2 h after treatment and remained even after 5 h. This priming effect correlates with previous studies were *in vivo* ASA administration led to physiological protection against ethanol cytotoxicity, such as reduction of protein carbonylation in plasma and PBMCs, as well as in TBARS from PBMCs [13]. The kinetics of these transient effects *in vivo* could be related with the pharmacodynamics of acetylsalicylic acid [22]. It’s interesting that the priming effect follows a similar kinetic pattern, been highest 2 hours after ASA challenge and disappearing after 5 hours.

In our study the effect of heat shock on expression of HSP-72 was tested by subjecting primary PBMCs from control or ASA-treated rats to an *in vitro* heat shock protocol. Our results suggest that ASA primes the heat shock response, which becomes fully active only after a second signal stress, such as *in vitro* heat shock. This study correlates with *in vivo* effects in central organs such as lung, liver, and kidney in ASA-treated animals [23]. Other studies in whole animals with ASA-treated rats have demonstrated metabolic effects in adipocytes [24]. Our results suggest that analyzing the effects of PBMCs could serve as an indicator of changes in central organs. To our knowledge, we are reporting for the first time that orogastric administration of ASA to rats primes PBMCs for HSP-72 expression. Lack of HSP-72 expression prior to heat shock could be important because, even at low levels, HSP-72 can affect diverse cellular processes, such as protein synthesis [25]. The EMSA of *in vivo* ASA treatment shows a 2 fold increase in the amount of HSF1 DNA-binding activity, suggesting an abundant amount of this transcription factor bound to chromatin in response to ASA. Interestingly, heat shock significantly decreased the amount of HSF1 resembling the levels found in cells from untreated animals (Figure 3A). It is possible that the abundant amount of HSF1 recruited to chromatin in response to ASA under in vivo conditions could explain the more efficient HSP72 expression both a mRNA and protein levels.

### Experiments *in vitro*

The idea that ROS, and H_2_O_2_ in particular, could serve as ASA mediators or second messengers has been postulated previously [26], [27], [28], [29]. In vascular endothelial cells, general stressors such as NO can promote translocation of HSF-1 to the nucleus [30]. Whether or not *in vivo* ASA promotes generation of H_2_O_2_ or some other ROS or NO precursors, our results support the notion that, in vitro, H_2_O_2_ in combination with ASA can mimic the priming effect induced by ASA *in vivo*.

ASA is recognized for mediating a variety of benefic effects *in vivo* that include a cytoprotective state, we have uncover that ASA primes the efficiency of HSP-72 expression in response to heat shock, which could contributes to cytoprotection.

## Materials and Methods

### Chemicals

Bovine serum albumin and ASA were obtained from the Sigma-Aldrich Co. (St. Louis, MO, USA). All remaining reagents were of the highest purity available. Bradford reagent was purchased from Bio-Rad Laboratories (Hercules, CA, USA).

### Animals

Male Wistar rats (200 g ±20 g, equivalent to 85 days) were fed a commercial diet (Nutricubos, Purina-Nestlé, Mexico City, Mexico), received water *ad libitum*, and were fasted for 16 h prior to treatment. The experimental animals were randomly divided into two groups and treated as follows: the control group received water through an orogastric tube and the experimental group received ASA through an orogastric tube (45 mg/kg Body weight [BW]). All animals were sacrificed 1, 2, 3 or 5 h after receiving ASA, blood samples were collected in heparinized tubes. This work was carried out following the rules under the Official Mexican Standards NOM-062-ZOO-1999 and the protocol was submitted to the Ethics Committee of the Faculty of Medicine, National Autonomous University of Mexico (UNAM), which approved the experimental protocol (ID 47-2007) under the responsibility of Dr Martha Zentella-de-Piña.

### Isolation of PBMCs

Immediately after obtaining the blood samples, PBMCs were isolated through a Lymphoprep™ (Axis-Shield, PoC AS, Oslo Norway) gradient according to manufacturer indications. Briefly, 5 ml of blood was diluted with isotonic Phosphate buffer saline solution (PBS) (1∶3). Nine ml of the diluted blood were added to a 15-ml conical tube containing 3 ml of Lymphoprep™. Diluted blood was added carefully to avoid mixing. After centrifugation at 1,800 x *g* for 30 min, the fraction that contained the PBMCs was carefully absorbed and washed with PBS.

### Heat Shock Protocol

The PBMCs were incubated in RPMI 1640 supplemented with 10% FBS and incubated at 42°C for 45 minutes. At the end of this heat shock, cells were allowed to recover by incubating them at 37°C for 2 hours. Aliquots were taken at different times, before heat shock (referred to as **T0**) immediately after heat shock (**HS**) or one (**1 h**) or two (**2 h**) hours after terminating heat shock (recovery time at 37°C). These aliquots were used for the extraction of total protein or total RNA for western blot or RT-PCR analysis.

### Nuclear Protein Extraction

Protein was obtained according to a previously described protocol [4].

### Protein Determination

Protein was determined as described by Bradford using a commercial solution (Biorad) [31].

### Extraction of Total RNA and RT-PCR RNA Assays

Total RNA was prepared from the PBMCs employing a commercial solution (Trizol™, Invitrogen). HSP-72 mRNA levels in the PBMCs were quantified by RT-PCR as previously described [32]. The amplification signal of HSP-72 was normalized with the amplification product for Glyceraldehyde-3-phosphatedehydrogenase mRNA (GAPDH), utilized as an endogenous internal standard. RT was performed on 1 µg of total RNA for 90 min at 42°C in a 5-µL reaction mixture containing 25 mmol/L Tris-HCl (pH 8.3), 50 mmol/L KCl, 5 mmol/L MgCl_2_, 2 mmol/L dithiothreitol, 1 mmol/L of the deoxynucleotides, 10 U avian myeloblastosis virus reverse transcriptase (Roche Molecular Biochemicals, Mannheim, Germany), 10 U ribonuclease inhibitor (Roche Molecular Biochemicals), and 0.8 µg oligo (dT)15 primer (Roche Molecular Biochemicals). RT was terminated by heating the sample at 95°C for 2 min.

Multiplexed PCR was carried out in a 20-µL reaction mixture containing 10 mmol/L Tris-HCl (pH 8.3), 50 mmol/L KCl, 1.5 mmol/L MgCl2, 2% dimethyl sulfoxide, 0.2 mmol/L of each deoxynucleotide, 0.1 mmol/L each of 5′ and 3′ GAPDH-specific primers, 1 mmol/L each of 5′ and 3′, HSP-72, specific primers, 25 ng of reverse-transcribed total RNA, and 0.5 U Taq DNA polymerase (Roche Molecular Biochemicals). HSP-72 sense primer: 5′GAGTCCTACGCCTTCAATATGAAG3′; HSP-72 antisense primer: 5′CATCAAGAGTCTGTCTCTAGCCAA3′. PCR amplification for HSP-72 was performed for 30 cycles, consisting of denaturation (94°C, 45 sec), annealing (60°C, 45 sec), and extension (72°C, 75 sec). After eight cycles (HSP-72), 0.1 mmol/L from each GAPDH primer was added to the reaction mixture and PCR cycles were continued. GAPDH sense primer: 5′CAGCAATGCATCCTGCAC3′; GAPDH antisense primer: 5′GAGTTGCTGTTGAAGTCACAGG3′. The PCR products were analyzed on 1% (w/v) agarose gels, stained with ethidium bromide, visualized with Ultraviolet (UV) trans-illumination, photographed, and submitted to image analysis.

### Mobility Shift Assay

DNA mobility shift assays were performed by employing a self-annealing HSE oligonucleotide probe (5′CTAGAAGCTTCTAGAAGCTTCTAG3′) as previously described [33]. DNA-binding reaction mixtures contained 10 µg of nuclear protein extract. The relative amount of protein concentration in all samples was determined utilizing commercial Bradford assays. Nuclear protein-extract volumes were adjusted so that equal amounts of protein were added to each binding reaction mixture. Binding reactions were performed with 1 mg of poly (dI-dC), 10 mM Tris (pH 7.8), 50 mM NaCl, 1 mM EDTA, 0.5 mM dithiothreitol, 5% glycerol in a final volume of 20 µl. Reaction mixtures were incubated at room temperature for 20 min and immediately loaded onto a 7.5% non-denaturing polyacrylamide gels containing 6.7 mM Tris-HCl (pH 7.5), 1 mM EDTA, and 3.3 mM sodium acetate. Gels were subjected to electrophoresis for 2.5 h at 150 V, dried, exposed to storage phosphor screen overnight, and revealed in a Typhoon 9400 (GE).

### Western Blot

Total protein extracts were fractionated by Sodium dodecyl sulfate-Polyacrylamide gel electrophoresis (SDS-PAGE) (10% acrylamide) and electro-blotted onto Polyvinylidene difluoride membranes (PVDF); the blots were blocked for 2 h at room temperature in TBST (20 mM Tris-HCl [pH 7.6], 137 mM NaCl, 0.1% [vol/vol] Tween 20) supplemented with 5% milk powder. Antibodies were diluted in TBST (1∶250 for anti-HSP-72 Ab). The blots were incubated in primary antibody for 2 h at room temperature. Blots were washed in TBST and incubated with the secondary Ab (horseradish peroxidase conjugated goat anti-mouse IgG (Bio-Rad), and diluted 1∶10,000 in TBST and 2.5% milk for 2 h at room temperature. Blots were washed, and proteins were visualized by chemiluminescence (Pierce). The luminescent signal was recorded in an autoradiography film (XAR; Kodak).

### Statistical Analysis

We used the SigmaStat 3.5 software for statistical analysis, statistical significance was established by applying the *t-studen*t test and significance was considered when P values were below 0.05.
